# Supplementary material for: A Novel KCNN2 Variant in a Family with Essential Tremor Plus: Clinical Characteristics and In Silico Analysis
Source: Genes (Basel). 2023 Jun 29;14(7):1380. doi: 10.3390/genes14071380 (PMC10379157; doi:10.3390/genes14071380)
Supplement: Supplementary file 1 [file genes-14-01380-s001.zip › genes-2433266-supplementary.pdf]

# Supplementary File S1

## List of genes associated to the disease:

A2M, AARS, AARS2, AASS, ABCB1, ABCB11, ABCB4, ABCB6, ABCB7, ABCC2, ABHD11, ABHD12, ACTL6B, ADA2, DAMTS13, ADARADCY5, AD, GRV1, ADH1A, ADH1B, ADH1C, ADPRHL2, ADRA2B, ADRB2, AFG3L2, AHI1, AIFM1, AKT1, ALAD, ALDH18A1, AMACR, ANO10, ANO3, ANOS1, AP2M1, AP3B2, APC2, APOB, APOE, APTX, AR, ARL13B, ARL3, ARMC9, ARNTL, ARSA, ARV1, ARX, ASAH1, ATCAY, ATG7, ATM, ATN1, ATP13A2, ATP1A2, ATP1A3, ATP2B3, ATP6AP2, ATP6V1A, ATP7B, ATP8B1, ATXN1, ATXN10, ATXN2, ATXN3, ATXN7, ATXN8, ATXN8OS, B9D1, BAZ1B, BCL7B, BCYRN1, BDNF, BEAN1, BRAT1, BSCL2, BTBD9, BUD23, C19orf12, C9orf72, CA8, CACNA1A, CACNA1B, CACNA1G, CACNA1H, CACNA1S, CAMTA1, CARS2, CASK, CBY1, CC2D2A, CCDC141, CCDC183, CCDC62, CCDC88C, CD28, CDH23, CDK19, CDKAL1, CDKL5, CDKN2B-AS1, CELF2, CEP104, CEP120, CEP126, CEP41, CFAP43, CHCHD10, CHCHD2, CHD2, CHD7, CIZ1, CLCN7, CLDN3, CLDN4, CLIP2, LN5, CLOCK, CLTC, CNKSR2, CNTN2, COA7, COL6A3, COMT, COQ2, COQ5, COQ8A, CP, CPLANE1, CRAT, CRHR1, CSPP1, CSTB, CTC1, CTDP1, CTH, CTLA4, CTNNA3, CTNND2, CTSF, CUL4B, CWF19L1, CYFIP2, CYP1A2, CYP27A1, CYP2C19, CYP2C8, CYP2C9, CYP2D6, DAB1, DALRD3, DARS2, DCC, DCTN1, DDOST, DHDDS, DLST, DMXL2, DNAJC13, DNAJC19, DNAJC30, DNAJC6, DNM1, DNM1L, DNMT1, DNMT3A, DPAGT1, DPM1, DRD2, DRD3, DRD4, DUSP6, EEF1A2, EIF4A1, EIF4G1, EIF4H, ELN, ELOVL4, ELOVL5, EPAS1, EPRS, ERCC2, ERCC3, ERCC4, ERCC5, ERCC6, ERCC8, ERLIN2, ETM1, ETM2, FAM126A, FAM149B1, FAR1, FARS2, FBXO7, FEZF1, FGF12, FGF13, FGF14, FGF17, FGF20, FGF8, FGFR1, FH, FKBP6, FLRT3, FLVCR1, FMR1, FRAXA, FRAXE, FRMPD4, FTL, FUS, FXN, GABARAP, GABRA1, GABRA2, GABRA3, GABRA4, GABRA5, GABRB2, GABRD, GABRE, GABRG2, GABRQ, GAK, GALC, GALT, GBA, GBA2, GCDH, GCH1, GCK, GGT1, GIGYF2, GJB1, GJC2, GLI2, GLIS3, GLUD2, GM2A, GNAL, GNAO1, GOSR2, GPAA1, GPR151, GPT2, GRIK2, GRIN2A, GRIN2B, GRIN2D, GRK5, GRM1, GSS, GSTP1, GTF2E2, GTF2H5, GTF2I, GTF2IRD1, GTF2IRD2, GTPBP2, HAPLN4, HAR1A, HCN1, HESX1, HIBCH, HLA-A, HLA-B, HLA-DQB1, HLA-DRB1, HMBS, HMGA2, HMOX1, HMOX2, HNF1A, HNF4A, HNMT, HPCA, HS1BP3, HS6ST1, HSPG2, HTR2A, HTR2C, HTRA2, HTT, HYLS1, IDH1, IFIH1, IFRD1, IL17A, IL17RD, IL1B, INPP5E, ITM2B, ITPA, ITPR1, JPH3, KCNA1, KCNA2, KCNB1, KCNC1, KCNC3, KCND3, KCNJ10, KCNJ18, KCNN2, KCNS2, , KIAA0556, KIAA0586, KIAA0753, KIF1B, KIF1C, KIF7, KISS1R, LAMA1, LAT2, LEMD3, LIMK1, LINGO1, LINGO2, LINGO4, LMNB1, LNPK, LRP12, LRPPRC, LRRK1, LRRK2, LYST, MAG, MAN1B1, MAOA, MAP2K5, MAPT, MARCH6, MARS, MATR3, MAX, MDH2, MECP2, MEIS1, MEN1, METTL27, MFN2, MICU1, MKS1, MLXIPL, MMAA, MMACHC, MME, MMP10, MORC2, MPLKIP, MPZ, MRE11, MRPL12, MSTO1, MTFMT, MTHFR, MTM1, MYH14, MYO5A, MYOT, NARS, NAXE, NCF1, NDNF, NDUFS2, NECAP1, NEMF, NEU1, NEUROD2, NEXMIF, NF1, NFASC, NGLY1, NIPA1, NKX2-1, NKX6-2, NMD3, NONO, NOP56, NOS1, NOS3, , NOTCH2NLC , NPHP1, NQO1, NR1H4, NR4A2, NSD1, NSMF, NTNG2, NTRK2, NUS1, NUTM2B-AS1, OCA2, OFD1, OGG1, OPA1, OPA3, OXR1, PAH, PANK2, PARK7, PARS2, PCBD1, PCDH19, PDE10A, PDE6D, PDGFB, PDGFRB, PDK3, PDYN, PER1, PEX10, PEX2, PEX6, PGAP1, PGK1, PIBF1, PIGN, PIGP, PIGQ, PIK3CA, PIK3R5, PINK1, PLA2G6, PLP1, PMP22, PMPCA, PNKP, PNP, PNPLA6, PODXL, POLG, POLG2, POLR1C, POLR3A, POLR3B, PON1, PPARGC1A, PPP1R15B, PPP1R1B, PPP2R2B, PPP3CA, PRDX1, PRICKLE1, PRKCG, PRKN, PRKRA, PRNP, PROK2, PROKR2, PRPS1, PSAP, PTEN, PTPRD, PTS, QDPR, QRICH1, RAB39B, RAB3GAP2, RARS, REEP1, REEP2, RET, RFC1, RFC2, RGS2, RIT2, RLIM, RNASEH2A, RNASEH2B, RNASEH2C, RNF113A, RNU12, RORA, RPGRIP1L, RPL10, RRM2B, SACS, SAMD12, SAMHD1, SATB1, SCARB2, SCN11A, SCN1A, SCN1B, SCN2A, SCN3A, SCN8A, SCN9A, SCP2, SCYL1, SDHA, SDHAF2, SDHB, SDHC, SDHD, SEMA3A, SETD2, SETX, SFXN4, SGCE, SIK1, SKOR1, SLC12A6, SLC13A5, SLC18A2, SLC1A2, SLC1A3, SLC20A2, SLC25A11, SLC25A13, SLC25A22, SLC25A4, SLC25A46, SLC2A1, SLC30A10, SLC39A14, SLC39A4, SLC52A2, SLC52A3, SLC5A7, SLC6A1, SLC6A17, SLC6A3, SLC6A4, SLC9A1, SLIT3, SMN1, SMN2, SNCA, SNCAIP, SNORD118, SNX10, SOD1, SOD2, SORT1, SOX10, SOX2-OT, SPEN, SPG11, SPR, SPRY4, SPTBN2, SQSTM1, SREBF1, ST3GAL5, STARD7, STK32B, STK39, STUB1, STX1A, STX1B STXBP1, SUFU, SYNGAP1, SYNJ1, SZT2, TACR3, TAF1, TARDBP, TARS, TAT, TBC1D24, TBL2, TBP, TCF7L2, TCIRG1, TCTN1, TCTN2, TCTN3, TENM4, TFG, TGM6, TH, THAP1, THOC2, TIMM8A, TK2, TMEM106B, TMEM127, TMEM216, TMEM237, TMEM240, TMEM270, TMEM67, TMEM70, TNFRSF1B, TNFSF11, TNNT1, TOR1A, TPI1, TPP1,

TRAK1, TRAPPC11, TRAPPC6B, TREX1, TRIM8, TRIO, TSFM, TSHR, TTC19, TTPA, TTR, TUBB4A, TWNK, UBA5, UBE3A, UCHL1, UFC1, UGT1A1, UGT2B7, UROC1, USP46, USP9Y, VAMP1, VAPB, VCP, VDR, VHL, VLDLR, VPS13A, VPS13C, VPS13D, VPS35, VPS37D, VWA3B, WARS2, WDR11, WDR45, WDR81, WFS1, WWOX, XPNPEP3, YEATS2, YWHAG, YY1, ZFR, ZFYVE26, ZNF142
